# Supplementary material for: Molecular noise of innate immunity shapes bacteria-phage ecologies
Source: PLoS Comput Biol. 2019 Jul 2;15(7):e1007168. doi: 10.1371/journal.pcbi.1007168 (PMC6629147; doi:10.1371/journal.pcbi.1007168)
Supplement: S1 Appendix — (PDF) [file pcbi.1007168.s001.pdf]

Molecular noise of innate immunity shapes bacteria-phage ecologies

Jakob Ruess, Maroš Pleška, Călin Guet and Gašper Tkačik

S.1 Self restriction in single cells and growing populations

In this section, we provide a more detailed description of self-restriction in single cells and demonstrate how a Markovian growth model for the population follows naturally under some mild assumptions.

Let N_S be the number of restriction sites on the DNA (i.e. the number of binding sites for R and M). Each of these sites can either be unmethylated (can be restricted or methylated), hemimethylated (can be further methylated), or double methylated (can be neither restricted nor methylated). In the following, we will denote the number of unmethylated, hemimethylated, and double methylated sites by S_0, S_1 and S_2, respectively. Assuming a well mixed reaction network and that it is equally likely that a molecule of M methylates an unmethylated or a hemimethylated site, it follows that the rates of restriction and methylation events are proportional to the number of available sites. Furthermore, we assume that no DNA-repair takes place and that any restriction event is lethal for the cell. With these mechanisms, the underlying stochastic process would be a finite state Markov chain with two absorbing states (all restriction sites double methylated or cell death) and more or less equivalent to the model that was used in [1] for calculating the probability of phage escape. However, in a growing cell population the DNA is continuously replicated with a speed that depends on the growth rate of the population and newly synthesized DNA is unmethylated. Given the double stranded nature of DNA and the mechanism of replication, when a double methylated restriction site gets replicated it will split into two hemimethylated sites, a hemimethylated site will split into a hemimethylated and an unmethylated site, and an unmethylated site will split into two unmethylated sites. This will double the total number of sites over the cell cycle. When the cell divides, daughter and mother will each receive N_S of the $2N_S$ sites. In order to not have to explicitly incorporate the cell cycle into the model, we average this over the cell cycle and assume that when a site splits into two sites in replication, with equal probability one of the new sites is kept by the cell whereas the other site is reserved for a future daughter cell and cannot cause self-restriction in the mother. This means that from the perspective of the mother cell, replication of a double methylated

site converts the site into a hemimethylated site, a hemimethylated site gets converted to an unmethylated site with probability 1/2 or stays hemimethylated with probability 1/2, and an unmethylated site stays unmethylated. Ignoring events that do not change the state of the system, we obtain the following model:

$$S_2 \underset{m}{\overset{\lambda}{\rightleftharpoons}} S_1 \underset{m}{\overset{\frac{1}{2}\lambda}{\rightleftharpoons}} \quad S_0 \xrightarrow{\ r\ } D, \tag{1}$$

where D stands for a single absorbing death state that stops the entire process. This stochastic process is a finite state Markov chain with $N_{\mathrm{S}}^3 + 1$ states. Using the conservation law $S_0 + S_1 + S_2 = N_{\mathrm{S}}$, the number of states that are actually needed to fully describe the chain is $((N_{\mathrm{S}}+1)^2 + N_{\mathrm{S}}+1)/2+1$, which means that the underlying master equation is a linear system of $((N_{\mathrm{S}}+1)^2 + N_{\mathrm{S}}+1)/2+1$ ordinary differential equations. Unfortunately, the total number of sites N_{S} typically ranges somewhere between $500 - 1000$, which means that determining the solution of the master equation would require us to compute the matrix exponential of a matrix whose size tends to be in the hundreds of thousands, which is difficult even if we only require a numerical solution. For a single site, however, we can set $N_{\mathrm{S}} = 1$ and it is straightforward to calculate with this model whose master equation is then given by the simple system of ordinary differential equations

$$\frac{\partial}{\partial t}\mathbf{p}(t) = \mathbf{A}\mathbf{p}(t), \text{ where } \mathbf{A} = \begin{bmatrix} 0 & r & 0 & 0 \\ 0 & -(r+m) & \lambda/2 & 0 \\ 0 & m & -(m+\lambda/2) & \lambda \\ 0 & 0 & m & -\lambda \end{bmatrix}, \tag{2}$$

and $\mathbf{p}(t) := [p_{\mathrm{D}}(t)\ p_0(t)\ p_1(t)\ p_2(t)]^\top$ with $p_{\mathrm{D}}(t) := \mathbb{P}(D(t) = 1)$ and $p_0(t)$, $p_1(t)$ and $p_2(t)$ as the probabilities for the site to be unmethylated, hemimethylated, or double methylated. Waiting times until self-restriction, such as displayed in Figure 2 in the main paper, can be readily derived from this equation using standard theory for finite state Markov chains [2].

In general, it becomes more difficult to obtain similar results when N_{S} is large, but we can make use of the fact that conditional on the chain not having reached the death state D the reaction network is a monomolecular reaction network whose master equation admits an analytical solution [3], which is a consequence of the implicit assumption that all restriction sites are equivalent and independent. With this in mind, and denoting by $S(t) = [S_0(t)\ S_1(t)\ S_2(t)]$ the unconditional chain, we can define the process $X(t) = S(t) \mid \{D(t) = 0\}$ and easily deduce that for all times t its distribution is a multinomial distribution

$$\mathbb{P}(X(t) = [x_0\ x_1\ x_2]) = \frac{N_{\mathrm{S}}!}{x_0!x_1!x_2!} p_{\mathrm{c},0}(t)^{x_0} p_{\mathrm{c},1}(t)^{x_1} p_{\mathrm{c},2}(t)^{x_2}, \tag{3}$$

where $p_{\mathrm{c},0}(t)$, $p_{\mathrm{c},1}(t)$ and $p_{\mathrm{c},2}(t)$ are the probabilities for a single site to be unmethylated, hemimethylated, or double methylated conditional on it not having been cut, respectively.

This allows us to analytically calculate with the full single cell model conditional on restriction not having happened in the cell, which turns out to be convenient for understanding self-restriction in growing populations where cells that cut themselves are removed from the population such that the internal methylation configurations of the still alive cells are determined by these conditional methylation-replication dynamics. Assuming that the dynamics in all cells are equilibrated, we can deduce that the internal methylation configuration of any randomly chosen cell at any time in a growing population follows the stationary distribution of $X(t)$, also known as the quasi stationary distribution of the unconditional absorbing Markov chain $S(t)$.

To obtain this quasi stationary distribution, and also the waiting time until self-restriction of cells in growing populations, we require the quasi stationary probabilities $p_{Q,0}, p_{Q,1}, p_{Q,2}$ for single sites. Writing the matrix $\mathbf{A}$ from Eq.(2) as

$$\mathbf{A} = \begin{bmatrix} 0 & \boldsymbol{c_1} \\ \mathbf{0} & \mathbf{C} \end{bmatrix} \text{ where } \boldsymbol{c_1} = [r \;\; 0 \;\; 0] \text{ and } \mathbf{C} = \begin{bmatrix} -(r+m) & \lambda/2 & 0 \\ m & -(m+\lambda/2) & \lambda \\ 0 & m & -\lambda \end{bmatrix},$$

we can obtain the quasi stationary distribution $\mathbf{p}_{\mathrm{QSD}} = [p_{\mathrm{Q},0} \; p_{\mathrm{Q},1} \; p_{\mathrm{Q},2}]^\top$ as the normalized right eigenvector of the transpose $\mathbf{C}'$ of $\mathbf{C}$ corresponding to the largest eigenvalue [4], i.e. as the solution of

$$\mathbf{C}'\mathbf{p}_{\mathrm{QSD}} = \gamma_1 \mathbf{p}_{\mathrm{QSD}}, \quad \text{s.t. } p_{\mathrm{Q},0} + p_{\mathrm{Q},1} + p_{\mathrm{Q},2} = 1$$

where γ_1 is the largest eigenvalues of $\mathbf{C}$. Due to the properties of $\mathbf{C}$, γ_1 is unique and guaranteed to be real and strictly negative if all reaction rates are positive $\lambda, r, m > 0$ (follows from the Perron-Frobenius theorem).

From a linear systems theory perspective the quasi stationary distribution can be understood as the distribution that equilibrates the system in all directions except the one leading into the absorbing state. A consequence is that if we use $\mathbf{p}_{\mathrm{QSD}}$ (and $p_{\mathrm{D}}(0) = 0$) as initial condition for the single site model, the solution of the master equation in Eq.(2) will evolve along a line in four dimensional space given by $\begin{bmatrix} 1 & -\mathbf{p}_{\mathrm{Q}}^\top \end{bmatrix}^\top$ towards the steady state given by $[1 \; 0 \; 0 \; 0]^\top$ which corresponds to probability one of the site being cut. These one-dimensional linear dynamics are the reason why the distribution of the waiting time τ for a single site to be cut has only one phase and reduces to an exponential distribution $\tau \sim Exp(-\gamma_1)$ as stated in the main paper. The waiting time τ_{S} until self-restriction of the cell is then obtained as the minimum of i.i.d. exponentially distributed random variables and is therefore also exponentially distributed $\tau_{\mathrm{S}} \sim Exp(-\gamma_1 N_{\mathrm{S}})$. The result that this waiting time follows an exponential distribution implies that growth of a self-restricting population can be modeled as a Markovian birth-death process, and that it makes sense to reason about a rate of self-restriction $\mu(r, m, \lambda)$ of a growing population.

In the main paper, we claimed that the self-restriction rate $\mu(r, m, \lambda)$ converges to zero as the enzyme activities converge to infinity, i.e. that $\lim_{r,m\mapsto\infty} (\mu(r, m, \lambda)) = 0$. It is

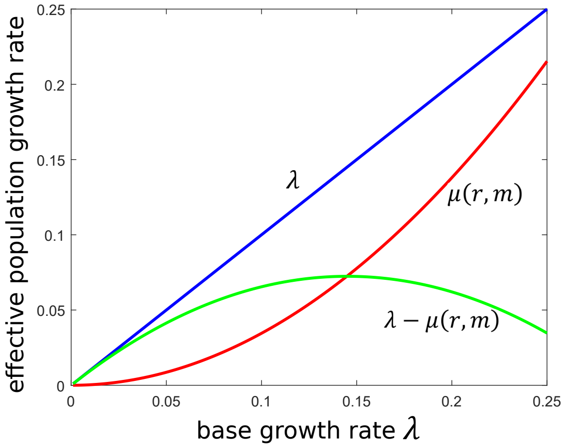

Fig A: Effective population growth rate as a function of the base growth rate λ for $N_S = 599$ restriction sites and fixed enzyme activities $m = 77.02/min$ and $r = 624.10/min$.

important to realize that for this to be true it is necessary but not sufficient that the probability that unmethylated restriction sites are ever available converges to zero. To validate this result, one needs to study the largest eigenvalue $\gamma_1 = \gamma_1(r, m, \lambda)$ of $\mathbf{C}$ as a function of r and m and take the limit of infinite enzyme activities. We tested this numerically by calculating $\gamma_1(r, m, \lambda)$ for growing values of r and m and found that $\gamma_1(r, m, \lambda)$, and hence $\mu(r, m, \lambda)$, indeed converges to zero as r and m become large. Interestingly, this result would not hold if also hemi-methylated restriction sites could be recognized by the restriction endonuclease. This can be seen by adding a restriction reaction for hemi-methylated sites to the model, and verifying that the largest eigenvalue of the corresponding matrix $\mathbf{C}$ does not converge to zero as r and m converge to infinity, despite the fact that the probability that hemi- or unmethylated restriction sites are ever available converges to zero. We conclude that bacteria can only prevent both self-restriction and phage escape at the same time because two subsequent demethylation steps need to take place to create susceptible sites, while any mechanism in which sites would become susceptible in a single step would retain a non-zero rate of self-restriction $\mu(r, m, \lambda)$ for large enzyme activities.

Finally, to supplement the analysis in Figure 3c of the main paper, we investigated numerically how the effective growth rate $\lambda_e(r, m, \lambda) = \lambda - \mu(r, m, \lambda)$ growth as a function of λ for a practically realistic scenario with $N_S = 599$ restriction sites and different fixed enzyme activities. The results (Figure A displays the case $m = 77.02/min$ and $r = 624.10/min$) suggest that there always exists a value of λ at which the self-restriction rate $\mu(r, m, \lambda)$ starts to grow superlinearly in λ, which implies that increasing λ further only decreases the effective growth rate of the population and that $\lambda_e(r, m, \lambda)$ will eventually drop to zero as λ increases (albeit this only happens for values of λ that are unrealistically large and not practically achievable for bacteria).

S.2 Stochastic simulation of self-restriction in growing populations

To verify the analytical results, as well as to evaluate the assumptions made in the main paper, we implemented a stochastic simulation algorithm for a growing population that implements our model of self-restriction and explicitly simulates every methylation, demethylation and restriction event in every cell. Growth of the population is represented as a stochastic birth process, i.e. the waiting time for a birth event in a population of size N is exponentially distributed $Exp(\lambda N)$. When a new cell is born, its initial methylation distribution is drawn from a multinomial distribution (see Eq.(3)) parametrized by the single site probabilities $\mathbf{p}_{\mathrm{new}} = [p_{\mathrm{new},0}\ p_{\mathrm{new},1}\ p_{\mathrm{new},2}]^\top$ with $p_{\mathrm{new},0}$, $p_{\mathrm{new},1}$ and $p_{\mathrm{new},2}$ as the probabilities for a single site to be unmethylated, hemi-methylated, or double methylated in the newly born cell. When a self-restriction event happens in any cell of the population, this cell is removed from the simulation.

Choosing $\mathbf{p}_{\mathrm{new}}$ as the quasi-stationary distribution (QSD) $\mathbf{p}_{\mathrm{QSD}}$ derived in the previous section corresponds to the assumptions made in the main paper and leads to perfect agreement between expected population sizes in the simulation and the analytical growth model in Eq.[3] in the main paper if the methylation patterns of all cells that are initially present in the population also follow the QSD (Figure B, panel A, black and blue). If the methylation patterns of the cells that are initially present do not follow the QSD (e.g. if all sites are fully methylated) but $\mathbf{p}_{\mathrm{new}}$ is chosen as the QSD, the methylation distribution of sites in the full population (including the initial cells) converges to the QSD (Figure B, panel B). In this case, since the methylation distribution of sites changes over time, so does the frequency of self-restriction events, implying that the mean population size does not follow a simple exponential (Figure B, panel A, green), but starts to resemble an exponential once convergence to the QSD is achieved. Setting $\mathbf{p}_{\mathrm{new}} = \mathbf{p}_{\mathrm{QSD}}$ is the only possibility that leads to a homogeneous population in which all cells follow the same methylation distribution at all times independently of their age. For any other choice of $\mathbf{p}_{\mathrm{new}}$ (e.g. all new cells start fully methylated), the population methylation distribution converges to a distribution different from $\mathbf{p}_{\mathrm{new}}$, even if all cells that are initially present also follow $\mathbf{p}_{\mathrm{new}}$ (Figure B, red growth curves).

If the RM-system is such that the number of restriction sites that it recognizes is relatively large, for instance $N_{\mathrm{S}} = 599$, while at the same time phage escape probability needs to be kept low, then according to our model it is necessary that also the enzyme activities are relatively large, for instance $m = 77.02$/min and $r = 624.10$/min for a growth rate $\lambda = 0.017$. Smaller values of r and/or m would imply either large self-restriction rates or phage escape probabilities (or both) since they would not allow to obtain a small number of cutting events at any of the $N_{\mathrm{S}} = 599$ bacterial restriction sites while ensuring at the same time that one of the typically small (e.g. $N_{\mathrm{V}} = 5$) number of restriction sites on the phage is reliably cut. Since the value of m is much larger than the growth rate $\lambda = 0.017$/min, which determines the demethylation rate of restriction sites, the QSD corresponding to these values of r and m is such that the probability for sites to be fully methylated is close to one while the probability for sites

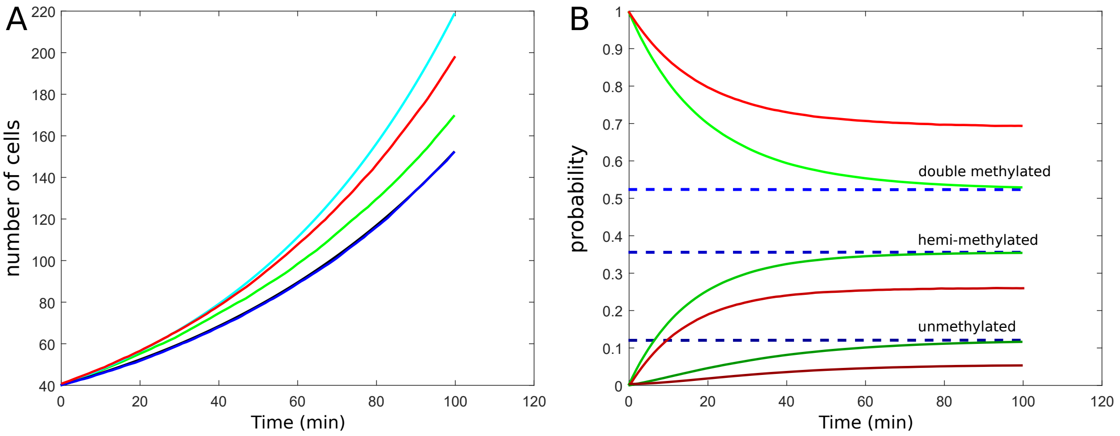

Fig B: **Stochastic simulation of population growth.** (A) Comparison of different growth curves. Cyan: exponential growth without self-restriction at rate λ. Black (matching with blue): exponential growth with self-restriction according to the analytical results in the main paper, i.e. at rate $\lambda - \mu(r, m, \lambda)$. Blue: expected population size in the stochastic growth simulation with $\mathbf{p}_{\text{new}} = \mathbf{p}_{\text{QSD}}$ and initial cells following $\mathbf{p}_{\text{QSD}}$. Green: expected population size in the stochastic growth simulation with $\mathbf{p}_{\text{new}} = \mathbf{p}_{\text{QSD}}$ and initial cells fully methylated. Red: expected population size in the stochastic growth simulation with $\mathbf{p}_{\text{new}} = [0\ 0\ 1]^\top$ and initial initial cells also following $\mathbf{p}_{\text{new}} = [0\ 0\ 1]^\top$. (B) Convergence of the population methylation distribution. Dashed blue lines: probabilities of finding restriction sites in the different configurations according to the QSD and in the growth simulation corresponding to the blue curve in A. Green colors: probability of finding restriction sites in the different configurations in the growth simulation corresponding the green curve in A. Red colors: probability of finding restriction sites in the different configurations in the growth simulation corresponding to the red curve in A. For all results of this figure we, chose $\lambda = 0.017/\text{min}, N_\text{S} = 599, m = 0.025/\text{min}, r = 5 \cdot 10^{-5}/\text{min}$, an intial population with 40 cells and performed $n = 200$ repetitions of each growth simulation to calculated expected population sizes.

to be empty is extremely low (in the order of 10^{-9}). Self-restriction happens nevertheless due to the large number of restriction sites as well as the large restriction rates which leads to essentially direct cutting in the (rare) cases when sites become unmethylated. A consequence of this is that the assumption that the methylation distribution of newly born cells follows the QSD is similar to assuming that new cells are initially fully methylated for these parameters. From a biological perspective it is unclear how accurate this assumption is since the precise dynamics of the real methylation-replication process are only poorly understood. Within our stochastic simulation, it is possible to test what consequences different assumptions about the methylation distribution of newly born cells have for growth of the population. For instance, instead of representing the replication of sites as demethylation events, we can explicitly carry out all replication events at division events of cells. This corresponds to assuming that demethylated sites are not generated continuously as in our model in the main paper, but can only be produced in bursts at cell division events and revert back to being fully methylated when the cells are not dividing. Under this assumption, the methylation pattern of cells depends on when the cell has divided for the last time and growing populations will never be homogeneous. Furthermore, the assumption that all restriction sites in a cell are replicated in a single event leads to loss of independence of individual sites, which, for the purpose of a simulation of this model, implies that choosing consistent methylation patterns for the cells that are present at time zero is a non-trivial problem and cannot be done by assuming some distribution for an individual site (e.g. $\mathbf{p}_{\mathrm{QSD}}$) and simply drawing all sites of all cells according to this distribution. Therefore, to obtain statistics for the initial cells that are consistent with the population dynamics, we first run a simulation with arbitrary initial methylation patterns for a time horizon that is sufficiently large to guarantee convergence of all population statistics. We then extract some cells ($N_0 = 40$ in plots) from the final population and use them as initial cells for a second simulation. The results (Figure C, panel A) show that this model can lead either to more or to less restriction events depending on the activities r and m of the enzymes. For the discussed parameters $\lambda = 0.017, N_{\mathrm{S}} = 599, m = 77.02/\mathrm{min}$ and $r = 624.10/\mathrm{min}$, for which the rate of methylation is more than three orders of magnitude larger than the growth rate, in most cases all restriction sites are re-methylated after division before the cell divides again and the population self-restriction rate is very small (Figure C, panel B).

S.3 Fluctuations in enzyme levels

In the main paper, we assumed that the enzyme activities m and r are the same in all cells. In practice, it may be hard and costly for cells to always keep m and r constant. In this section, we supplement the results of the main paper by investigating the consequences of fluctuations in enzyme activities. In particular, we study how populations grow if different cells have different enzyme activities. For now, let us assume that

(i) The values of r and m vary between cells but remain approximately constant within each cell over its entire lifetime.

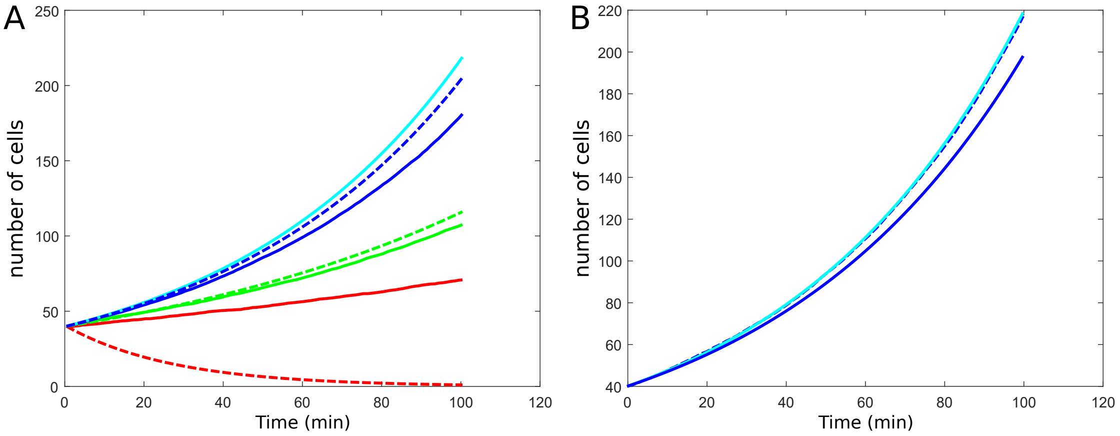

Fig C: **Stochastic simulation of population growth under different model assumptions.** (A) Comparison of expected population sizes (obtained by averaging over $n = 100$ simulation runs) in the two models for parameters $N_0 = 40, \lambda = 0.017/\text{min}, N_S = 599, m = 0.25/\text{min}$ and $r = 0.05/\text{min}$ (red), $r = 0.005/\text{min}$ (green), $r = 0.0005/\text{min}$ (blue), and $r = 0$ (cyan, growth without self-resriction). Solid lines: population growth with splitting of sites at cell division. Dashed lines: population growth according to the model of the main paper. (B) Comparison of expected population sizes (obtained by averaging over $n = 100$ simulation runs) in the two models for parameters: $N_0 = 40, \lambda = 0.017/\text{min}, N_S = 599, m = 77.02/\text{min}$ and $r = 624.1/\text{min}$.

(ii) The values of r and m in a newly born daughter cell are drawn independently from the values of its mother cell according to a fixed distribution $p_{\mathrm{R,M}}$.

(iii) The population is growing much faster than it is self-restricting.

Under these assumptions, it is straightforwards to extend the results of the main paper to the case where m and r follow a distribution over the population and to show that the expected population size $n(t)$ of such a population would follow the growth model

$$\frac{d}{dt}n(t) = (\lambda - \mathbb{E}\left[\mu(R, M, \lambda)\right])\, n(t), \text{ where} \tag{4}$$

$$\mathbb{E}\left[\mu(R, M, \lambda)\right] = \int_m \int_r \mu(r, m, \lambda) p_{\mathrm{R,M}}(r, m) dr dm, \text{ and}$$

$$\mu(r, m, \lambda) = \mathbb{E}\left[\tau_{\mathrm{S}}\right]^{-1} = \frac{N_{\mathrm{S}}}{-\mathbf{p}_{\mathrm{QSD}}\mathbf{B}^{-1}\mathbf{1}} = -\gamma_1 N_{\mathrm{S}}. \tag{5}$$

This implies that the population self restriction rate can simply be obtained by averaging $\mu(r, m, \lambda)$ over the population distribution $p_{\mathrm{R,M}}(r, m)$ of enzyme activities. Similarly, we can calculate a population phage escape probability by averaging $p_{\mathrm{V}}(r, m)$ over $p_{\mathrm{R,M}}(r, m)$ and ask how these quantities change when the variability in enzyme activities between cells increases. For $\mu(r, m, \lambda)$, we can understand this as imposing the distribution $p_{\mathrm{R,M}}(r, m)$ onto Figure 3b in the main paper and averaging the displayed values over this distribution. More or less independently of the precise shape of $p_{\mathrm{R,M}}(r, m)$, we find that increasing the marginal variances of $p_{\mathrm{R,M}}(r, m)$ increases both the population self-restriction rate and phage escape probability, but that the increase in self restriction is small as long as the mean activity of M is large compared to its variance and the growth rate λ while the increase in phage escape probability is small if the distribution $p_{\mathrm{R,M}}(r, m)$ is such that the enzyme activities are positively correlated.

With this established, we can return to the conditions (i)-(iii) under which these results are valid and ask what violation of the assumptions would imply for growth of the population. Condition (i) allows us to calculate the survival time of each cell with fixed values of r and m using Eq.(4) in the main paper. This condition is necessary because random fluctuations in time in r and m within a cell would affect the waiting time distribution until self-restriction and the detailed mechanisms governing these fluctuations would have to be known. One exception to this is when r and m fluctuate much faster than the typical time to self-restriction. In this case one could make use of time scale separation arguments [5] and replace r and m by their expectations because fast fluctuations simply average over all possible values.

Conditions (ii) and (iii) ensure that the distribution of r and m in the population remains approximately constant in time despite the fact that cells with different values of r and m survive for different times. It is interesting, and also useful for clarification, to study how the population would grow if these conditions are violated. It is easy to see that if condition (ii) is violated, for instance if cells inherit similar values of r and m from their

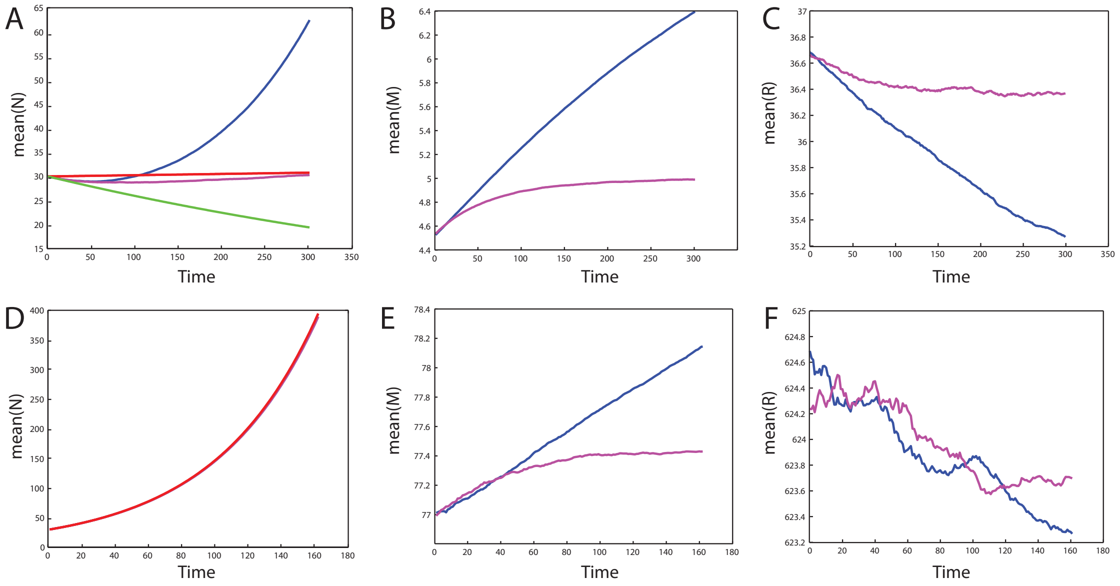

Fig D: **Study of population growth ($\lambda = 0.017$/min) with variability in R and M.** For all results, we chose the distribution $p_{\mathrm{R,M}}$ assuming independence of R and M with both marginals being log-normal distributions with coefficient of variation 0.3. Red: Growth without variability, i.e. with self-restriction rate $\mu(\mathbb{E}[R], \mathbb{E}[M])$. Green: Growth with variability according to assumptions (i)-(iii), i.e. with self-restriction rate $\mathbb{E}[\mu(R, M)]$. Magenta: Stochastic simulation (mean of 10,000 runs) of a population where new cells get new random values of r and m. Blue: Stochastic simulation (mean of 10,000 runs) of a population where new cells inherit almost the same values of r and m from their mothers. (A-C) Population with very large self-restriction rate ($N_{\mathrm{S}} = 599$, $\mathbb{E}[M] = 4.53$/min, $\mathbb{E}[R] = 36.71$/min). (D-F) Population with low self-restriction ($N_{\mathrm{S}} = 599$, $\mathbb{E}[M] = 77.02$/min, $\mathbb{E}[R] = 624.10$/min). (A,D) Average population size. (B,E) Average value of m in the population. (C,F) Average value of r in the population.

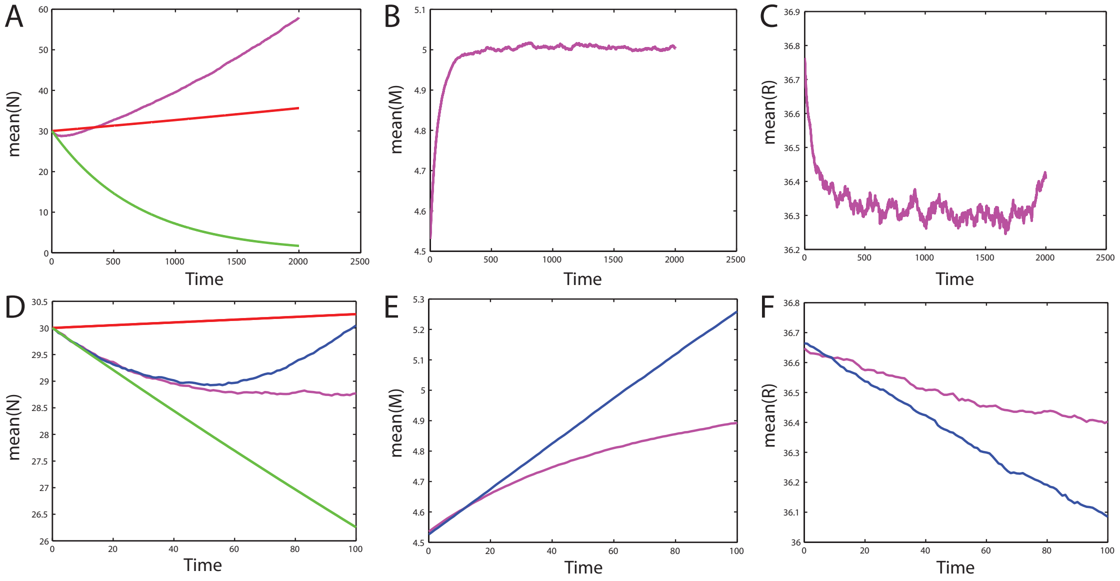

Fig E: **Detailed study of the population with large self-restriction rate of Figure D.** Color coding is the same as in Figure D. (A-C) Long-term dynamics. (D-F) Short term dynamics.

mother cells, then as the population grows large values of m would get enriched in the population over time. A bit less obvious, yet nevertheless true, is that in populations that self-restrict approximately as fast as they grow, m would still get enriched over time even if new cells get entirely new random values of r and m. This is because cells are still "selectively killed" based on their values of r and m. This induces a bias to large values of m and small values of r which is counteracted by the birth of new cells with random values of r and m, equivalently to the biased methylation configuration distributions discussed in Figure 3a in the main paper. Consequently, there should exist a quasi stationary distribution to which the values of r and m in a growing population converge.

To investigate conditions (ii) and (iii) in detail, we performed a numerical study and implemented a stochastic simulation algorithm that can be used to simulate population growth when (ii) and (iii) are violated and allows for a freely tunable distribution $p_{\mathrm{R,M}}$ according to which new values of r and m are assigned to newly born cells. This distribution can be adjusted to incorporate different scenarios, i.e. it could be chosen such that new cells receive random values of r and m or such that they can only obtain values of r and m that were already present in the population (inheritance of the values from the mother cell). Figure D and Figure E show simulation results for different scenarios and different parameter values. First, we investigated a population that is barely growing because the self-restriction rate is almost as large as the growth rate (Figure D,

panels (A)-(C)). In particular, we chose $\lambda = 0.017$/min, $N_S = 599$, $\mathbb{E}[M] = 4.53$/min, $\mathbb{E}[R] = 36.71$/min and the distribution $p_{R,M}$ assuming independence of R and M with both marginals being log-normal distributions with coefficient of variation 0.3. The red line shows the expected population size without variability in enzyme activities, i.e. when the self-restriction rate is $\mu(\mathbb{E}[R], \mathbb{E}[M]) = 0.0169$/min. With variability in R and M, the population self-restriction rate becomes $\mathbb{E}[\mu(R, M)] \approx 0.0183$/min under assumptions (i)-(iii) and the expected population shrinks according to the green line. Blue and magenta show the results of 10,000 runs of the stochastic simulation algorithm where we assumed that either new cells obtain completely randomized values of r and m according to $p_{R,M}$ (magenta) or new cells inherit similar values from their mothers (blue). The simulation results show that initially the population size shrinks with the same slope as the green line but over time cells with lower self-restriction rates become enriched in the population and growth exceeds even the growth rate of the noise free population. A close-up view of the early dynamics is provided in Figure E (panel D). It follows that under these assumptions variability in enzyme levels leads to initially smaller growth rates but eventually allows the population to grow faster. Figure E (panels A-C) shows the long-term dynamics of the population where new cells obtain completely randomized values of r and m according to $p_{R,M}$. It can be seen that in this scenario the means of R and M in the population converge to constant values, i.e. the population eventually achieves a constant growth rate. Panels (D-F) in Figure D provide the same study as panels (A-C) for a population with a small self-restriction rate ($\lambda = 0.017$/min, $N_S = 599$, $\mathbb{E}[M] = 77.02$/min, $\mathbb{E}[R] = 624.1$/min, $p_{R,M}$ chosen log-normal as in panels (A-C)). It can be seen that there are almost no differences between all the growth models in this case.

S.4 Bacteria-phage ecology

In this section, we study alternative ecological scenarios and prove the results in Table 1 in the main paper. While a comprehensive investigation of all plausible ecological scenarios is out of scope of this paper, we do want to stress that the framework for quantifying the cost of immunity that we developed in this paper provides a general and easy to evaluate functional relationship between the single cell details of RM-systems and a population level growth cost. It is therefore straightforward to connect this framework to any standard model [6, 7, 8, 9] of phage ecology and to study how the cost of immunity might affect bacteria-phage dynamics. In this paper, we limit ourselves to studying the consequences of the different efficiency criteria for bacteria colonizing phage dominated environments that are listed in the main paper. Furthermore, we provide simulation results for two simple models, tracking either only bacterial population growth or the ecological dynamics of methylated and non-methylated phages interacting with a bacterial population comprised of cells with and cells without RM-systems.

S.4.1 A minimal model of bacterial population dynamics under constant phage load

To gain some first insights into the dynamics of a bacterial population, $n(t)$, under constant phage load, v, we consider a simplistic model that combines bacterial growth and reduction of the population size through phage infections in a single ordinary differential equation:

$$\frac{\mathrm{d}n(t)}{\mathrm{d}t} = \lambda_{\mathrm{e}}(r, m, k, \lambda) \cdot n(t) - \frac{\rho v}{l} p_{\mathrm{V}}(r, m, k) \cdot n(t)^2. \tag{6}$$

The first term on the right-hand side describes the growth of the population at an effective rate of $\lambda_{\mathrm{e}}(r, m, k, \lambda)$ and already accounts for self-restriction as detailed in the main paper. The second term accounts for phage escape events. Here, v is the (unit-free) size of the phage population which we take to be a constant parameter of the environment; phages enter bacterial cells at a rate proportional to $vn(t)$, as prescribed by mass-action kinetics, with a proportionality constant given by the phage adsorption rate, ρ. The rate of successful infections is then given by $\rho v p_{\mathrm{V}}(r, m, k)n(t)$. We assume that each successful infection event wipes out a fraction, $0 < \frac{1}{l} < 1$, of the total population (i.e., on average, $n(t)/l$ bacteria die following phage escape), yielding the full Eq (6). For simplicity, we assume all infections to be lytic and do not consider the possibility of the phage to lysogenize the host bacteria.

In this minimal model, two key quantities summarize the fate of bacterial populations in the presence of bacteriophages: λ_e quantifies the short-term growth rate before rare but potentially catastrophic phage escape events are likely to occur, while the long-term cost of phage escape can be quantified through the steady state population size to which the population can maximally grow for given v and a given configuration of the RM-system (see Figure F, panel (a)). It can be shown that the one biologically-relevant fixed point of the growth dynamics that represents the steady-state bacterial population size is given by

$$n_s(r, m, k, \lambda) := \frac{n(t \to \infty)}{l} = \frac{\lambda_e(r, m, k, \lambda)}{\rho v p_{\mathrm{V}}(r, m, k)}, \tag{7}$$

where division by l in the definition of $n_s(r, m, k, \lambda)$ is incorporated here to point out that the steady state of this model is the same as the expression for the "expected growth until phage escape" that we found in Table 1 in the main paper up to a constant that does not depend in any way on the single-cell parameters r, m, and the number of concurrently active RM-systems, k. We can conclude that model Eq.(6) suggests to measure bacterial performance in the same way as scenario (i) in the main paper, and that analyzing trade-offs according to this model would lead to the same results as the main paper but additionally allows to interpret points on the provided Pareto fronts as possible trajectories of bacterial growth dynamics. To illustrate this, Figure F, panel (b), shows growth curves in which λ_e is fixed to half the base growth rate λ and the RM-systems are optimized to maximize $n_s(r, m, k, \lambda)$ given this λ_e for different number

of concurrently active RM-systems, k. It can be seen that $k_{\text{opt}} = 2$ is the optimal number of RM-systems to grow at the this rate λ_e.

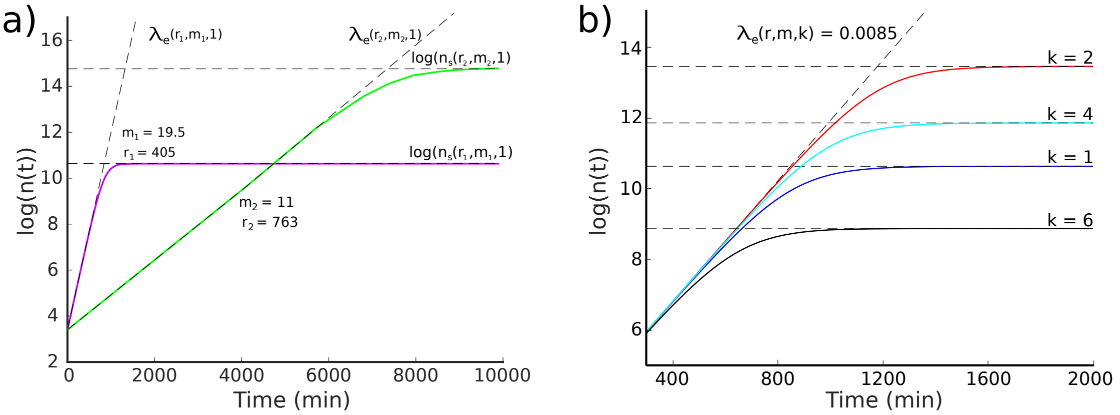

Fig F: **Bacterial growth for a minimal ecological model.** Parameters were assumed to be $N_S = 599$, $N_V = 5$, and $\lambda = 0.017\ \text{min}^{-1}$ in line with the main paper and the strain used in [10] carrying the EcoRI system. Furthermore we fixed $c = 10^{-5}$ and $l/\rho v = 1$ min. **(a)** Two possible growth curves, Eq (6), for bacteria with a single RM-system and different enzyme activities (in min^{-1}). **(b)** Growth curves leading to the maximally possible steady state values at fixed effective growth rate, $\lambda_e = 0.5\lambda = 8.5 \cdot 10^{-3}\ \text{min}^{-1}$, for different k show that intermediate values of k (here, $k_{\text{opt}} = 2$) optimize the steady-state population size.

S.4.2 A coupled model of bacteria-phage ecology

To obtain a plausible model of the coupled ecological dynamics of phages interacting with bacteria carrying RM-systems, we start from the model in reference [6] where Campbell considered a simple phage predator model in a chemostat. In particular, Campbell's model comprises the phage concentration $v(t)$ and the bacterial concentration $n(t)$ evolving according to the following equations:

$$\frac{\mathrm{d}n(t)}{\mathrm{d}t} = \lambda n(t)\left(1 - \frac{n(t)}{L}\right) - \rho v(t)n(t) - an(t)$$
$$\frac{\mathrm{d}v(t)}{\mathrm{d}t} = \rho N\left[n(t)v(t)\right] - \rho n(t)v(t) - k_I v(t) - av(t), \tag{8}$$

where a is the flow rate of the chemostat, ρ is the phage adsorption rate, k_I the rate of spontaneous phage inactivation, N the number of phages that are produced when a bacterium is infected. The growth law is assumed to be logistic with growth rate λ and maximal level L. Note that to be in line with Campbell's model, $n(t)$ and $v(t)$ are defined as concentrations in this section while elsewhere in the paper population sizes

are generally treated as unit-free quantities. Consequently, also the parameters in this section have different units than in the rest of the paper. Furthermore, we point out that the original formulation of Campbell's model included a delay explicitly modeling the time that it takes from infection of a cell to lysis and the release of new phages. However, using such a delay in a chemostat model would require one to also take into account that some infected cells are washed out of the chemostat before they are lysed and release phage [7]. To keep our models easily understandable for readers not familiar with this literature, we have decided to omit the delay in our simulations.

Eq.(8) captures the dynamics of phages interacting with bacteria that do not have a RM-system and has the property that, depending on the model parameters, the phage either dies out when it grows too slowly compared to the flow rate and the rate of phage inactivation or that it grows sufficiently fast and keeps the bacteria at a very low level [6]. To expand this model with bacteria that contain RM-systems, we need to add (at least) two additional species to it: bacteria that have RM-systems $n_r(t)$ and phages $v_r(t)$ whose restrictions sites are already methylated because they have been produced as the result of a phage escape event on RM$^+$-bacteria. If methylated or normal phages infect bacteria that do not have a RM-system, the newly produced phages are unmethylated because the methyl-transferase is not present in the cell in which the new phages are produced. Overall, our expanded model is comprised of the following equations:

$$
\begin{aligned}
\frac{\mathrm{d}n(t)}{\mathrm{d}t} &= \lambda n(t)\left(1-\frac{n(t)+n_r(t)}{L}\right)-\rho n(t)\left(v(t)+v_r(t)\right)-an(t)\\
\frac{\mathrm{d}n_r(t)}{\mathrm{d}t} &= \lambda_e n_r(t)\left(1-\frac{n(t)+n_r(t)}{L}\right)-\rho n_r(t)\left(p_V v(t)+v_r(t)\right)-an_r(t)\\
\frac{\mathrm{d}v(t)}{\mathrm{d}t} &= \rho N n(t)\left(v(t)+v_r(t)\right)-\rho v(t)\left(n(t)+n_r(t)\right)-k_I v(t)-av(t)\\
\frac{\mathrm{d}v_r(t)}{\mathrm{d}t} &= \rho N n_r(t)\left(v_r(t)+p_V v(t)\right)-\rho v_r(t)\left(n_r(t)+n(t)\right)-k_I v_r(t)-av_r(t), \qquad (9)
\end{aligned}
$$

where all parameters have the same meaning as in Campbell's model and $p_V = p_V(r, m, k)$ and $\lambda_e = \lambda_e(r, m, k)$ are phage escape probability and effective population growth rate of RM$^+$ bacteria as defined in the main paper. For the sake of simplicity, we assume that bacteria have either no RM-system or that they have all k RM-systems (or alternatively that $k = 1$) since otherwise additional bacterial and phage species would need to be tracked in the model. For a first simulation of this model, we assumed that $\lambda = 0.017\mathrm{min}^{-1}$, $\rho = 0.001\mathrm{ml/min}$, $k_I = 0.0001\mathrm{min}^{-1}$, $a = 0.005\mathrm{min}^{-1}$, $L = 1/\mathrm{ml}$, $N = 100$, and that the RM-systems are not very costly but also not very efficient with $p_V = 10^{-3}$ and $\lambda_e = 0.016\mathrm{min}^{-1}$. Figure G shows short and long-term dynamics of the system starting from an initial condition where bacterial and phage concentrations are low and methylated phages are completely absent. It can be seen that initially, due to the low phage concentration, both RM$^-$ and RM$^+$ bacteria grow almost unaffected by phages. Phages, on the other hand, do not grow but are maintained in the environment due to a small number of infections on RM$^-$ bacteria that approximately balance the

flow rate of the chemostat and phage inactivation. At some point, however, when the population of RM$^-$ bacteria has grown to a sufficiently large size, the production of new phages surpasses the loss of phages. This leads to an increasing phage population and more infections, and consequently causes the concentration of RM$^-$ bacteria to drop quickly. With less RM$^-$ bacteria to prey upon also the phage population plummets again. However, the increased number of phages leads to phage escape events on RM$^+$ bacteria and the production of methylated phages. These methylated phages then start to prey upon the RM$^+$ bacteria leading to prey-predator damped oscillatory dynamics between these two populations. On a long time horizon the system equilibrates to a steady state where the environment contains mostly methylated phages while both RM$^-$ and RM$^+$ bacteria are maintained at low levels (Figure G, panel b).

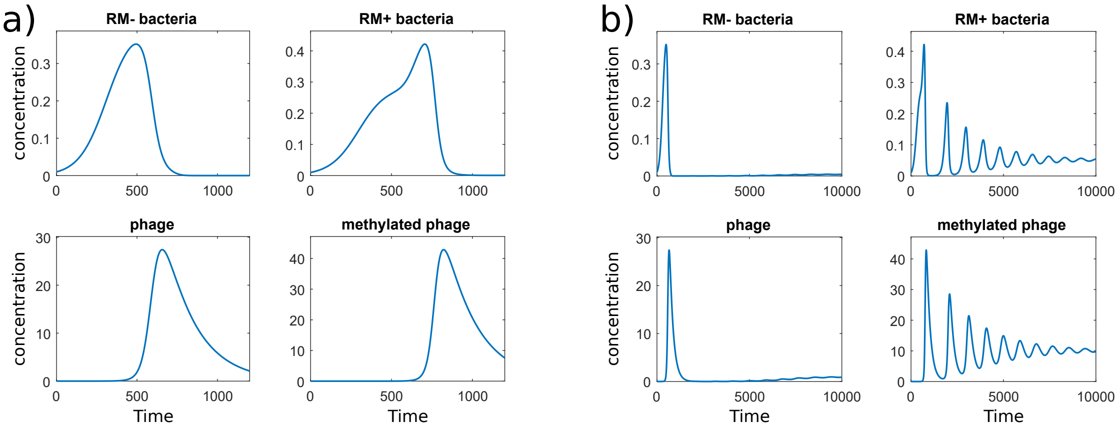

Fig G: **Bacteria-phage ecological dynamics for inefficient RM-systems.** Phage escape and effective growth rate have been chosen as $p_V = 10^{-3}$ and $\lambda_e = 0.016\text{min}^{-1}$. The initial condition for the system was chosen as $n(0) = 0.011$/ml, $n_r(0) = 0.011$/ml, $v(0) = 0.011$/ml, and $v_r(0) = 0$. **(a)** Short-term dynamics. **(b)** Long-term dynamics.

To see whether more efficient RM-systems allow bacteria to defend themselves more successfully, we performed another simulation of the model assuming $p_V = 10^{-10}$ and $\lambda_e = 0.016\text{min}^{-1}$. Figure H shows that the dynamics of the system remain overall similar with the difference that the reduced phage escape probability delays the time when the population of methylated phages starts to increase. It does not, however, prevent methylated phages from eventually taking over and the steady state population sizes of bacteria remain at equally low levels as before. Decreasing the phage escape probability even further does not change anything about this result. To back up this result, assuming the same RM-system characteristics as in the main paper ($N_S = 599$, $N_V = 5$, $c = 3.744 \cdot 10^{-7}$) and $k = 1$, we calculated effective population growth rate $\lambda_e(r, m, k)$ and phage escape probability $p_V(r, m, k)$ for 1.25 million parameter combinations of r and m (on a 2-dimensional grid up to $r = 50000\text{min}^{-1}$ and $m = 200\text{min}^{-1}$) according to

the equations provided in the main paper. For each parameter combination, we used a numerical solver to approximately calculate the steady state of the model in Eq.(9) and found that in all cases the steady state concentration of RM^+ bacteria remains below 0.11/ml, that is below approximately 10% of the maximally possible concentration L in the logistic growth model. We conclude that, at least according to the considered deterministic model in which phages cannot go completely extinct, RM-systems can only provide transient resistance against phages and that the efficiency of RM-systems would have to be quantified as the duration of this transient resistance, similar to the scenarios studied in Table 1 in the main paper.

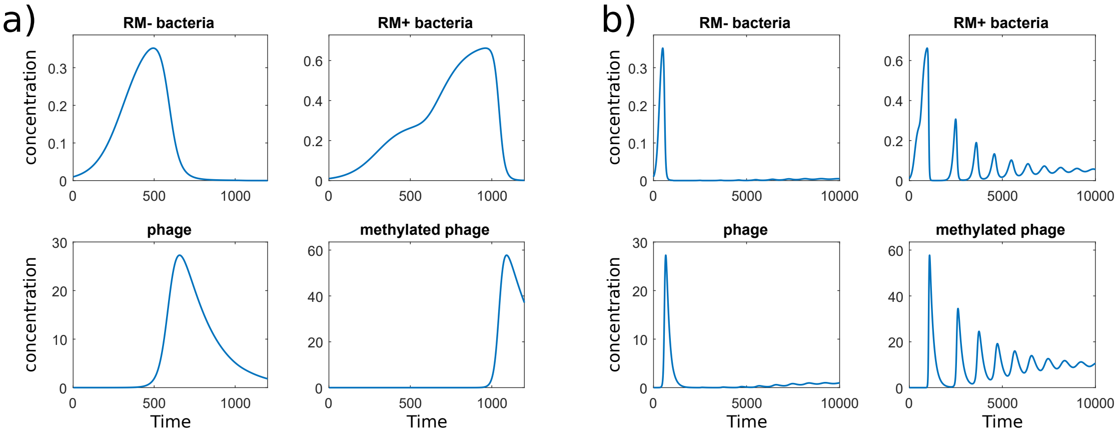

Fig H: **Bacteria-phage ecological dynamics for efficient RM-systems.** Phage escape and effective growth rate have been chosen as $p_V = 10^{-10}$ and $\lambda_e = 0.016\text{min}^{-1}$. The initial condition for the system was chosen as $n(0) = 0.011/\text{ml}$, $n_r(0) = 0.011/\text{ml}$, $v(0) = 0.011/\text{ml}$, and $v_r(0) = 0$. **(a)** Short-term dynamics. **(b)** Long-term dynamics.

S.4.3 Proof of the results in Table 1 of the main paper

Theorem. Consider a bacterial population $n(t)$ of initial size $n(0) = n_0$ growing exponentially at rate λ_e according to $n(t) = e^{\lambda_e t} n_0$ in a well-stirred environment containing a number of phages v that remains constant at all times. Let c_{mut} be the rate of the occurrence of immunity conferring mutations per cell and unit time, $\rho_v := \rho \cdot v \cdot p_V$ where ρ is the phage adsorption rate and p_V the phage escape probability. If we denote by τ_p

the time of the first phage escape event, it holds that

$$(a) \qquad \mathbb{E}[n(\tau_p)] = \begin{cases} n_0 + \frac{\lambda_e}{\rho_v} & \text{if } \lambda_e > 0 \\ n_0 + \frac{\lambda_e\left(1-e^{\frac{n_0\rho_v}{\lambda_e}}\right)}{\rho_v} & \text{if } \lambda_e < 0. \end{cases}$$

$$(b) \qquad \mathbb{E}\left[\int_0^{\tau_p} c_{\text{mut}} n(t)dt\right] = \begin{cases} \frac{c_{\text{mut}}}{\rho_v} & \text{if } \lambda_e > 0 \\ \frac{c_{\text{mut}}}{\rho_v} + c_{\text{mut}} e^{\frac{n_0\rho_v}{\lambda_e}} \left(n_0 - \frac{1}{\rho_v}\right) & \text{if } \lambda_e < 0. \end{cases}$$

Proof:
In a well-stirred environment with constant number of phages v, the rate of phages infecting bacteria is given by $\rho \cdot v \cdot n(t)$ according to mass action kinetics. The rate of successful infections is then $\delta(t) := \rho_v n(t)$ and the occurrence of successful infections can be regarded as firings of a non-homogeneous Poisson process with rate $\delta(t)$. It follows from the theory of non-homogeneous Poisson processes that the probability density function f_{τ_p} of τ_p is given by

$$f_{\tau_p}(t) = \delta(t)e^{-\eta(t)}, \quad \text{where}$$
$$\eta(t) = \int_0^t \delta(s)ds = \int_0^t \rho_v n(s)ds = \rho_v \int_0^t e^{\lambda_e s} n_0 ds = \frac{\rho_v n_0}{\lambda_e}\left(e^{\lambda_e t} - 1\right).$$

It follows that the density function can be expressed as

$$f_{\tau_p}(t) = \rho_v n_0 \exp\left\{\frac{\rho_v n_0}{\lambda_e} + \lambda_e t - \frac{\rho_v n_0}{\lambda_e} e^{\lambda_e t}\right\}. \tag{10}$$

To prove (a), writing out the expectation and plugging in Eq. (10) yields

$$\begin{aligned}
\mathbb{E}[n(\tau_p)] &= \int_0^\infty n(t) f_{\tau_p}(t)dt \\
&= \int_0^\infty e^{\lambda_e t} n_0 \rho_v n_0 \exp\left\{\frac{\rho_v n_0}{\lambda_e} + \lambda_e t - \frac{\rho_v n_0}{\lambda_e} e^{\lambda_e t}\right\} dt \\
&= n_0^2 \rho_v \int_0^\infty \exp\left\{\frac{\rho_v n_0}{\lambda_e} + 2\lambda_e t - \frac{\rho_v n_0}{\lambda_e} e^{\lambda_e t}\right\} dt \\
&= n_0^2 \rho_v \frac{1}{n_0^2 \rho_v^2}\left\{\lambda_e + n_0\rho_v - e^{\frac{n_0\rho_v}{\lambda_e}} \lim_{t\to\infty} g(t)\right\} \quad \text{where}
\end{aligned}$$
$$g(t) = \exp\left\{-\frac{n_0\rho_v e^{\lambda_e t}}{\lambda_e}\right\}\left(\lambda_e + n_0\rho_v e^{\lambda_e t}\right).$$

Using $n_0 > 0$ and $\rho_v > 0$, it follows that

$$\lim_{t\to\infty} g(t) = \begin{cases} 0 & \text{if } \lambda_e > 0 \\ \lambda_e & \text{if } \lambda_e < 0, \end{cases}$$

and thus

$$\mathbb{E}\left[n(\tau_p)\right] = \frac{1}{\rho_v}\left(\lambda_{\mathrm{e}} + n_0\rho_v - e^{\frac{n_0\rho_v}{\lambda_{\mathrm{e}}}}\lambda_{\mathrm{e}}\mathbb{1}_{\{\lambda_{\mathrm{e}}<0\}}\right)$$

$$= \begin{cases} n_0 + \frac{\lambda_{\mathrm{e}}}{\rho_v} & \text{if } \lambda_{\mathrm{e}} > 0 \\ n_0 + \frac{\lambda_{\mathrm{e}}\left(1-e^{\frac{n_0\rho_v}{\lambda_{\mathrm{e}}}}\right)}{\rho_v} & \text{if } \lambda_{\mathrm{e}} < 0, \end{cases}$$

which concludes the proof of (a).

To prove (b), writing out the expectation and plugging in Eq. (10) yields

$$\mathbb{E}\left[\int_0^{\tau_p} n(t)dt\right] = \int_0^{\infty}\left\{\int_0^{s} n(t)dt\right\} f_{\tau_p}(s)ds$$

$$= \int_0^{\infty}\left\{\int_0^{s} e^{\lambda_{\mathrm{e}}t}n_0 dt\right\}\rho_v n_0 \exp\left\{\frac{\rho_v n_0}{\lambda_{\mathrm{e}}} + \lambda_{\mathrm{e}}s - \frac{\rho_v n_0}{\lambda_{\mathrm{e}}}e^{\lambda_{\mathrm{e}}s}\right\}ds$$

$$= n_0^2\rho_v \int_0^{\infty} \frac{e^{\lambda_{\mathrm{e}}s}-1}{\lambda_{\mathrm{e}}} \exp\left\{\frac{\rho_v n_0}{\lambda_{\mathrm{e}}} + \lambda_{\mathrm{e}}s - \frac{\rho_v n_0}{\lambda_{\mathrm{e}}}e^{\lambda_{\mathrm{e}}s}\right\}ds$$

$$= \frac{n_0^2\rho_v}{\lambda_{\mathrm{e}}}\int_0^{\infty}\left\{\exp\left\{\frac{\rho_v n_0}{\lambda_{\mathrm{e}}} + 2\lambda_{\mathrm{e}}t - \frac{\rho_v n_0}{\lambda_{\mathrm{e}}}e^{\lambda_{\mathrm{e}}t}\right\} - \exp\left\{\frac{\rho_v n_0}{\lambda_{\mathrm{e}}} + \lambda_{\mathrm{e}}t - \frac{\rho_v n_0}{\lambda_{\mathrm{e}}}e^{\lambda_{\mathrm{e}}t}\right\}\right\}ds$$

$$= \frac{n_0^2\rho_v}{\lambda_{\mathrm{e}}}\frac{1}{n_0^2\rho_v^2}\left\{\lambda_{\mathrm{e}} + n_0\rho_v - e^{\frac{n_0\rho_v}{\lambda_{\mathrm{e}}}}\lim_{t\to\infty} g(t)\right\} + \frac{n_0^2\rho_v}{\lambda_{\mathrm{e}}}\frac{1}{n_0\rho_v}\left\{\lim_{t\to\infty} h(t) - 1\right\}, \quad \text{where}$$

$$h(t) = \exp\left\{\frac{n_0\rho_v - n_0\rho_v e^{\lambda_{\mathrm{e}}t}}{\lambda_{\mathrm{e}}}\right\}.$$

Using $n_0 > 0$ and $\rho_v > 0$, it follows that

$$\lim_{t\to\infty} h(t) = \begin{cases} 0 & \text{if } \lambda_{\mathrm{e}} > 0 \\ e^{\frac{n_0\rho_v}{\lambda_{\mathrm{e}}}} & \text{if } \lambda_{\mathrm{e}} < 0, \end{cases},$$

and thus

$$\mathbb{E}\left[\int_0^{\tau_p} n(t)dt\right] = \frac{1}{\lambda_{\mathrm{e}}\rho_v}\left(\lambda_{\mathrm{e}} + n_0\rho_v - e^{\frac{n_0\rho_v}{\lambda_{\mathrm{e}}}}\lambda_{\mathrm{e}}\mathbb{1}_{\{\lambda_{\mathrm{e}}<0\}}\right) + \frac{n_0}{\lambda_e}e^{\frac{n_0\rho_v}{\lambda_{\mathrm{e}}}}\mathbb{1}_{\{\lambda_{\mathrm{e}}<0\}} - \frac{n_0}{\lambda_e}$$

$$= \begin{cases} \frac{1}{\rho_v} & \text{if } \lambda_{\mathrm{e}} > 0 \\ \frac{1}{\rho_v} + e^{\frac{n_0\rho_v}{\lambda_{\mathrm{e}}}}\left(n_0 - \frac{1}{\rho_v}\right) & \text{if } \lambda_{\mathrm{e}} < 0. \end{cases}$$

which concludes the proof of (b) since c_{mut} is just a multiplicative constant.

The theorem proves the results for the efficiency criteria (i) and (iii) in Table 1 of the main paper. To see why $\mathbb{P}\left(\tau_{\mathrm{mut}} < \tau_p\right) = \frac{c_{\mathrm{mut}}}{c_{\mathrm{mut}}+\rho_v}$ for criterion (ii), we note that mutation events can be represented firings of an inhomogenous Poisson processes with a

rate $c_{\text{mut}}n(t)$ that has the same dependence on the population size as the rate $\rho_v n(t)$ of successful infection events. As a consequence, the probability that mutation happens before phage escape is simply the same as the probability that a homogenous Poisson process with rate c_{mut} fires before another homogenous Poisson process with rate ρ_v, i.e., equal to $\frac{c_{\text{mut}}}{c_{\text{mut}}+\rho_v}$.

Corollary 1. If the effective growth rate of the population is positive, the expected growth of the bacterial population until phage escape, the probability that mutation happens before phage escape, and the expected integrated population mutation rate until phage escape are all independent of the initial size of the population. The probability that mutation happens before phage escape and the population mutation rate are furthermore independent of the bacterial growth rate.

Corollary 2. For all three bacterial performance criteria in Table 1 in the main paper, the optimal bacterial defense strategy is constant in time.

Proof: This follows directly from the result that the performance criteria are independent of the initial population size. In particular, if a population is growing and phage escape has not happened up to a certain time t_1, then the population size will have changed from $n(0)$ to $n(t_1)$. If the efficiency of immunity as quantified by the three bacterial performance criteria in Table 1 in the main paper would depend on population size, then the optimal bacterial defense strategy would need to be adjusted for the new population size $n(t_1)$. Given that this is not the case, however, we find that there exist a single best defense strategy that does not change when the population is growing.

Since Corollary 2 has interesting biological implications, we will in the following discuss further for case (i) in Table 1 in the main paper how the expected amount by which the bacterial population can grow before phage escape, $\mathbb{E}[n(\tau_p)] - n_0$ becomes independent of the initial population size n_0. In fact, as we will demonstrate below one can also show the stronger results that $g(\tau_p) := n(\tau_p) - n_0$ follows an exponential distribution with parameter $\frac{\rho_v}{\lambda_e}$ that is independent of n_0, despite the seemingly explicit dependence on n_0 and the fact that the probability density function of τ_p does depend on n_0. To see this, let us first denote the probability density function of $g(\tau_p)$ by $f_{g(\tau_p)}(n)$, where we have chosen n as the running variable of the density to indicate that this is a probability density over population sizes. $f_{g(\tau_p)}(n)$ can be obtained from the probability density function of τ_p by exploiting the change of variables formula

$$f_{g(\tau_p)}(n) = \frac{d}{dn}\left(g^{-1}(n)\right) \cdot f_{\tau_p}\left(g^{-1}(n)\right) \tag{11}$$

where g^{-1} is the inverse function of g. Since $g(t) = n(t) - n_0 = e^{\lambda_e t}n_0 - n_0$ we obtain

its inverse function as

$$g^{-1}(n) = \frac{1}{\lambda_e} \log\left(\frac{n+n_0}{n_0}\right) \tag{12}$$

To evaluate Eq.(11), we note that

$$\frac{d}{dn}\left(g^{-1}(n)\right) = \frac{1}{\lambda_e(n+n_0)} \tag{13}$$

while the second term can be obtained by plugging $g^{-1}(n)$ into Eq.(10):

$$\begin{aligned} f_{\tau_p}\left(g^{-1}(n)\right) &= \rho_v n_0 \exp\left\{\frac{\rho_v n_0}{\lambda_e}\left(1-\frac{n+n_0}{n_0}\right) + \log\left(\frac{n+n_0}{n_0}\right)\right\} \\ &= \rho_v n_0 \frac{n+n_0}{n_0} \exp\left\{\frac{\rho_v}{\lambda_e}\left(n_0-(n+n_0)\right)\right\} \\ &= \rho_v(n+n_0)\exp\left\{-\frac{\rho_v}{\lambda_e}n\right\} \end{aligned} \tag{14}$$

Plugging Eq.(13) and Eq.(14) into Eq.(11) gives

$$f_{g(\tau_p)}(n) = \frac{\rho_v}{\lambda_e} e^{-\frac{\rho_v}{\lambda_e}n}, \tag{15}$$

which can be recognized as the probability density function of an exponential distribution with parameter $\frac{\rho_v}{\lambda_e}$ and hence mean $\frac{\lambda_e}{\rho_v}$, as already derived earlier. We can conclude that the entire probability distribution of $g(\tau_p) = n(\tau_p) - n_0$ is independent of n_0. Correspondingly, for any bacterial performance measure that is derived from this probability distribution, such as the mean in criteria (i) in Table 1 in the main paper, one will obtain the result that the optimal bacterial defense strategy against phages does not depend on the bacterial population size.

To visualize this mathematical result graphically, we fixed parameters $\rho v = 1\text{min}^{-1}$, and $\lambda_e = 0.016\text{min}^{-1}$ and $p_V = 1.6 \cdot 10^{-5}$ in line with experimental results in [10] obtained for the EcoRI restriction modification system and calculated growth curves and all relevant probability densities using different values for initial population sizes. Figure I, panel (a), shows how two different initial conditions ($n_0 = 10$ and $n_0 = 100$) yield populations that require different amounts of time to grow to large sizes, but also how phages tend to escape faster in the case where the population starts at $n_0 = 100$. Panel (b) then visualizes how these two effects cancel each other out and how the probability distribution of the population increase until phage is the same in both cases.

S.4.4 Pareto optimality for efficiency criteria (ii) and (iii) in Table 1 of the main paper.

In this section, we investigate how the Pareto optimal fronts in Figure 4 of the main paper change when efficiency criteria (ii) or (iii) in Table 1 of the main paper are considered instead of efficiency criterion (i). Accordingly, using the same parameters as in panels

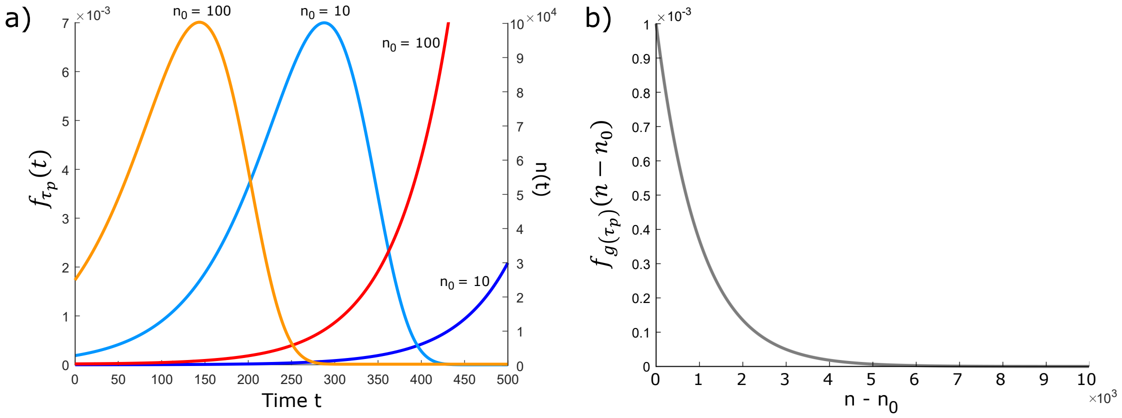

Fig I: **Population growth until phage escape is independent of population size.** Parameters have been chosen as $\rho v = 1\text{min}^{-1}$, and $\lambda_e = 0.016\text{min}^{-1}$ and $p_V = 1.6 \cdot 10^{-5}$. **(a)** Exponential growth curves starting from initial population sizes $n_0 = 10$ (blue) and $n_0 = 100$ (red). The corresponding waiting time distributions on phage escape show that phage escape is likely to occur very early in the case where the population starts at $n_0 = 100$ (orange) while in the case where the population starts at $n_0 = 10$ (light blue) the population reaches large sizes much later but phage escape also happens later. **(b)** The two opposing effects visualized in (a) cancel each other such that the probability distribution of the population increase until phage escape, $n(\tau_p) - n_0$ becomes independent of n_0 and given by an exponential distribution with parameter $\frac{\rho_v}{\lambda_e}$.

c and d in Figure 4 of the main paper, and additionally assuming that the per cell mutation rate is given by $c_{\text{mut}} = 10^{-8}\text{min}^{-1}$, we calculated Pareto optimal fronts between $\lambda_e(r, m, k)$ and $\mathbb{P}(\tau_{\text{mut}} < \tau_p)$ for criterion (ii) and $\mathbb{E}\left[\int_0^{\tau_p} c_{\text{mut}} n(t) dt\right]$ for criterion (iii). The results are displayed in Figure J. The results for efficiency criterion (iii) are overall relatively similar to the results with criterion (i) of the main paper. This is because the Pareto fronts are mostly shaped by the functional dependency of the criteria on the phage escape probability, which is the same in the two cases. In criterion (ii), on the other hand, the bacterial performance is quantified as $\mathbb{P}(\tau_{\text{mut}} < \tau_p) = \frac{c_{\text{mut}}}{c_{\text{mut}} + \rho_v}$ and the dependence on the phage escape probability is therefore more complex than just plain division through $p_{\text{V}}(r, m, k)$ as was the case for the two other criteria. For criterion (ii), we find that the main question is whether the rate of mutation c_{mut} is a larger or smaller than $\rho_v = \rho \cdot v \cdot p_v(r, m, k)$. If c_{mut} is larger than ρ_v, the probability that mutation happens before phage escape is typically close to one. If c_{mut} is smaller the probability is typically close to zero. There also exists an intermediate regime in which c_{mut} and ρ_v are about the same size, or at least about the same order of magnitude, such that the probability for mutation to happen before phage escape is substantial but smaller than one. With the exception of the case where cells have only a single RM-system ($k = 1$), however, this regime is relatively small. Consequently, for any given mutation rate c_{mut}

there exists a minimal RM-system protection from phages that is necessary to ensure that $\mathbb{P}(\tau_{\mathrm{mut}} < \tau_p) \approx 1$ and a tuning of the RM-system(s) that realizes just this minimal protection while ensuring that the effective growth rate λ_e is not reduced any more than is necessary. This optimal tuning is, however, a function of the environment given that $\rho_v = \rho \cdot v \cdot p_{\mathrm{V}}(r, m, k)$ depends on the number of phages v. For the parameter values considered here, we find that $k = 3$ RM-systems can ensure nearly full protection with the smallest reduction in effective growth rate ($\lambda_e^{\mathrm{opt}} \approx 0.015\mathrm{min}^{-1}$, see black cross in Figure J, panel (a)).

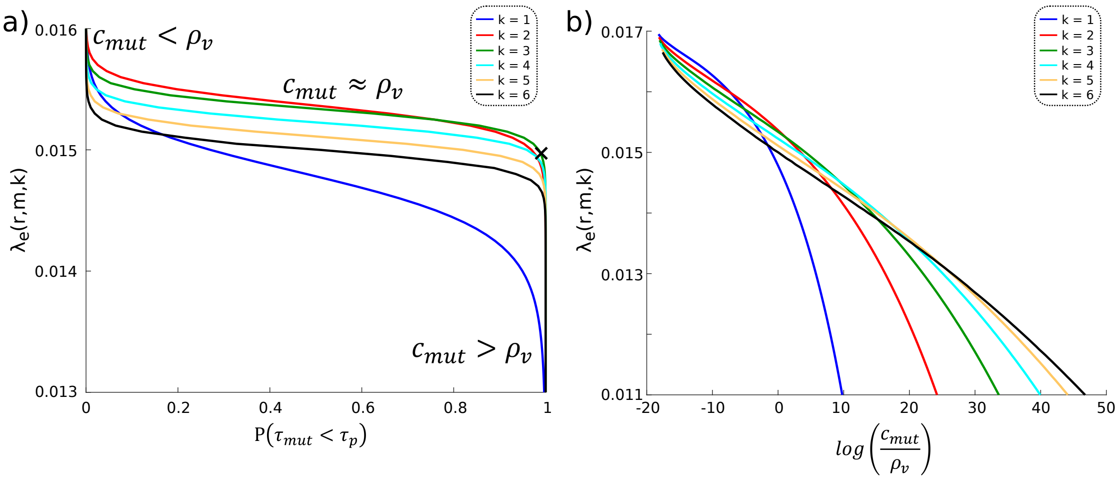

Fig J: **Optimal tradeoff in the presence of phages.** For both panels, we set $N_S = 599$, $N_V = 5$, $\lambda = 0.017\mathrm{min}^{-1}$, $c = 3.744 \cdot 10^{-7}$, $c_{\mathrm{mut}} = 10^{-8}\mathrm{min}^{-1}$, and $\rho \cdot v = 1\mathrm{min}^{-1}$. **(a)** Pareto optimal fronts for efficiency criterion (ii). The black cross marks the maximal possible effective growth rate that allows near perfect protection from phages. **(b)** Pareto optimal fronts for efficiency criterion (iii).

S.4.5 Optimal regulation of enzyme expression levels

To supplement the analysis in the main paper, we investigated in more detail how the optimal enzyme activities change along the Pareto fronts in Figure 4b in the main paper, that is, how different trade-offs between λ_e and n_s are realized for different numbers of systems k. The results (Figure K) suggest that any Pareto-optimal enzyme activities $m_{k,opt}$ and $r_{k,opt}$ are necessarily such that the growth cost $\lambda - \lambda_e$ is allocated in equal parts to self-restriction and metabolic cost. A consequence is that both the optimal total combined enzyme activity and the optimal total self-restriction rate are fully determined by the specific trade-off and do not depend on k, i.e. that

$$k \cdot c \cdot (m_{k,opt} + r_{k,opt}) = c \cdot (m_{1,opt} + r_{1,opt}) = \mu(r_{1,opt}, m_{1,opt}) = k \cdot \mu(r_{k,opt}, m_{k,opt}) = \frac{\lambda - \lambda_e}{2}.$$

Phrased differently, this result can also be understood as follows: for every trade-off, the metabolic cost parameter c fixes the total allowed enzyme activity to $\frac{\lambda-\lambda_e}{2c}$. This enzyme activity then needs to be optimally allocated to restriction and modification taking into account the number of RM-systems k.

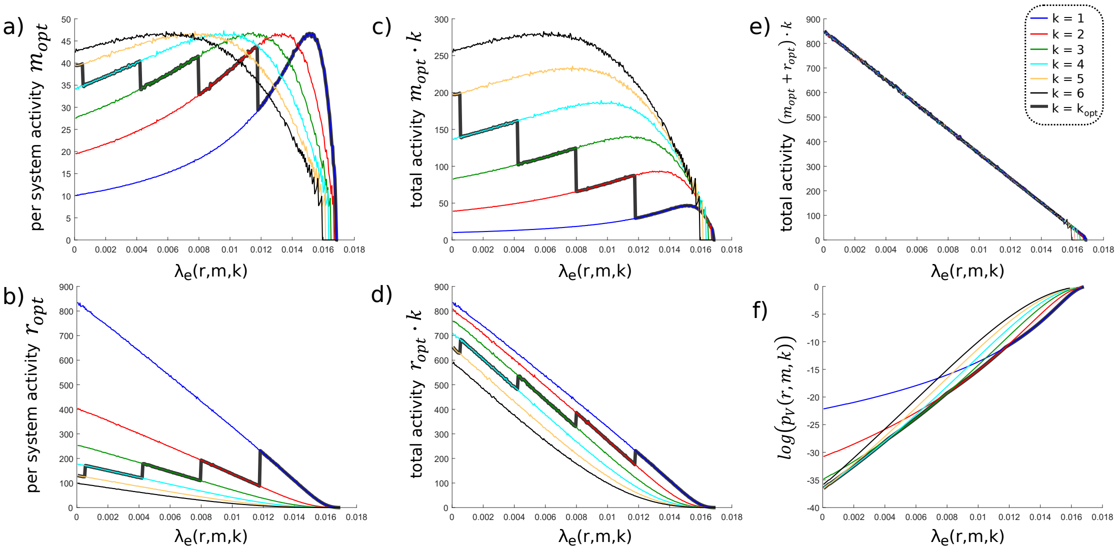

Fig K: **Detailed study of optimal enzyme activities.** Optimal enzyme activities, r_{opt} and m_{opt} (in $\min^{-1}$), on Pareto fronts for different k, as a function of effective growth rate, $\lambda_e(r, m, k)$. **(a,b)** Same as Figure 4c in the main paper. Additionally, the case $k = 5$ is shown (yellow) and it is outlined which system is optimal at which effective growth rate (thick black). **(c,d)** Same as panel a and b but showing the total enzyme activities $r_{opt} \cdot k$ and $m_{opt} \cdot k$. **(e)** Optimal total combined activity $(m_{opt} + r_{opt}) \cdot k$ of restriction and modification enzymes as a function of $\lambda_e(r, m, k)$. **(f)** Logarithm of optimal phage escape probabilities $p_V(r, m, k)$ as a function of $\lambda_e(r, m, k)$.

References

[1] F. Enikeeva, K. Severinov, and M. Gelfand. Restriction-modification systems and bacteriophage invasion: Who wins? *Journal of Theoretical Biology*, 266:550–559, 2010.

[2] P. Buchholz, J. Kriege, and I. Felko. *Input modeling with phase-type distributions and Markov models.* Springer Briefs in Mathematics, Berlin, Germany, 2014.

[3] T. Jahnke and W. Huisinga. Solving the chemical master equation for monomolecular reaction systems analytically. *Journal of Mathematical Biology*, 54(1):1–26, 2007.

[4] J. Darroch and E. Seneta. On quasi-stationary distributions in absorbing continuous-time finite markov chains. *Journal of Applied Probability*, 4(1):192–196, 1967.

[5] C. Rao and A. Arkin. Stochastic chemical kinetics and the quasi-steady-state assumptions: Application to the gillespie algorithm. *The Journal of Chemical Physics*, 118(11):4999, 2003.

[6] A. Campbell. Conditions for the Existence of Bacteriophage. *Evolution*, 15(2): 153–165, 1961.

[7] B. Levin, F. Stewart, and L. Chao. Resource-Limited Growth, Competition, and Predation: A Model and Experimental Studies with Bacteria and Bacteriophage. *The American Naturalist*, 111(977):3–24, 1977.

[8] R Korona and BR Levin. Phage-mediated selection and the evolution and maintenance of restriction-modification. *Evolution*, 47(2):556–575, 1993.

[9] T. Frede Thingstad. Elements of a theory for the mechanisms controlling abundance, diversity, and biogeochemical role of lytic bacterial viruses in aquatic systems. *Limnology and Oceanography*, 45(6):1320–1328, 2000.

[10] M. Pleška, L. Qian, R. Okura, T. Bergmiller, Y. Wakamoto, E. Kussell, and C. Guet. Bacterial Autoimmunity Due to a Restriction-Modification System. *Current Biology*, 26(3):404–409, 2016.

Table A: **List of parameters.** In this table, we summarize the model parameters used in the main text and their roles in our work. Some numerical values that have been used at different places of the manuscript are provided. We point out, however, that the main text is not based on numerical simulations of the employed models and that we needed to specify concrete numerical values for only some of the parameters.

**Parameter name**	**letter**	**appearance**	**role in the manuscript**	**units**	**(exemplary) value(s)**
cellular restriction rate	r	single cell model	optimization variable	inv. time	free and $1.2 \cdot 10^3$/min
cellular methylation rate	m	single cell model	optimization variable	inv. time	free and $1.5 \cdot 10^2$/min
growth rate	λ	single cell model	different values considered	inv. time	0.017/min, Figs 3c,4c
# bact. restr. sites	N_S	single cell model	given by RM system	none	599 (Fig 4) and 5 (Fig 3)
# of phage restr. sites	N_V	phage escape model	given by RM system and phage	none	5
# of RM systems	k	growth and phage esc.	given by the bacterial strain	none	$1-6$
cost of enzyme expression	c, c_r,c_m	bact. growth model	different values considered	none	$3.7 \cdot 10^{-7}$, Figs 4a,d
phage escape probability	p_V	ecology models	given by r, m, k, and N_V	none	e.g. $\in [10^{-2}, 10^{-8}]$
effective growth rate	λ_e	ecology models	given by λ, r, m, k, N_S, c_r, c_m	inv. time	between 0 and λ
phage adsorption rate	ρ	ecology models	does not affect trade-offs	inv. time	none
initial bacterial pop. size	n_0	ecology models	does not affect trade-offs	none	none
(initial) phage pop. size	v	ecology models	does not affect trade-offs	none	none
fraction of killed bacteria	l	ecology model (o)	does not affect trade-offs	none	none
mutation rate per bacteria	c_{mut}	ecology models (ii,iii)	used for supplementary results	inv. time	10^{-8}/min
